# Supplementary material for: Dead cells release a ‘necrosignal’ that activates antibiotic survival pathways in bacterial swarms
Source: Nat Commun. 2020 Aug 19;11:4157. doi: 10.1038/s41467-020-17709-0 (PMC7438516; doi:10.1038/s41467-020-17709-0)
Supplement: Supplementary file 1 — Supplementary Information [file 41467_2020_17709_MOESM1_ESM.pdf]

Supplementary Figures and Tables

for

Dead cells release a necrosignal that activates antibiotic survival pathways in bacterial swarms

Souvik Bhattacharyya, David M. Walker, and Rasika M. Harshey

Department of Molecular Biosciences

University of Texas at Austin

Austin, TX 78712

Supplementary Figures

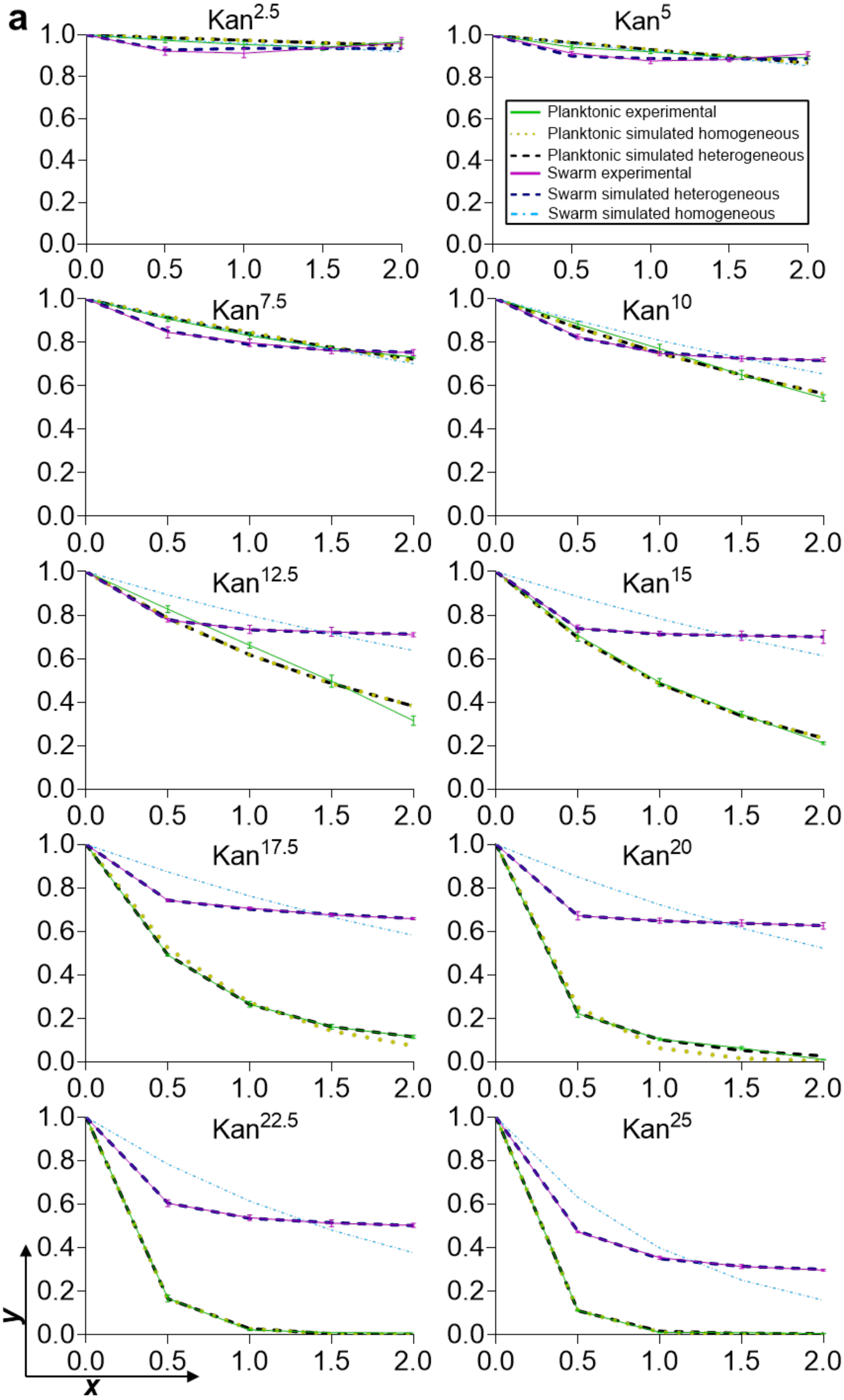

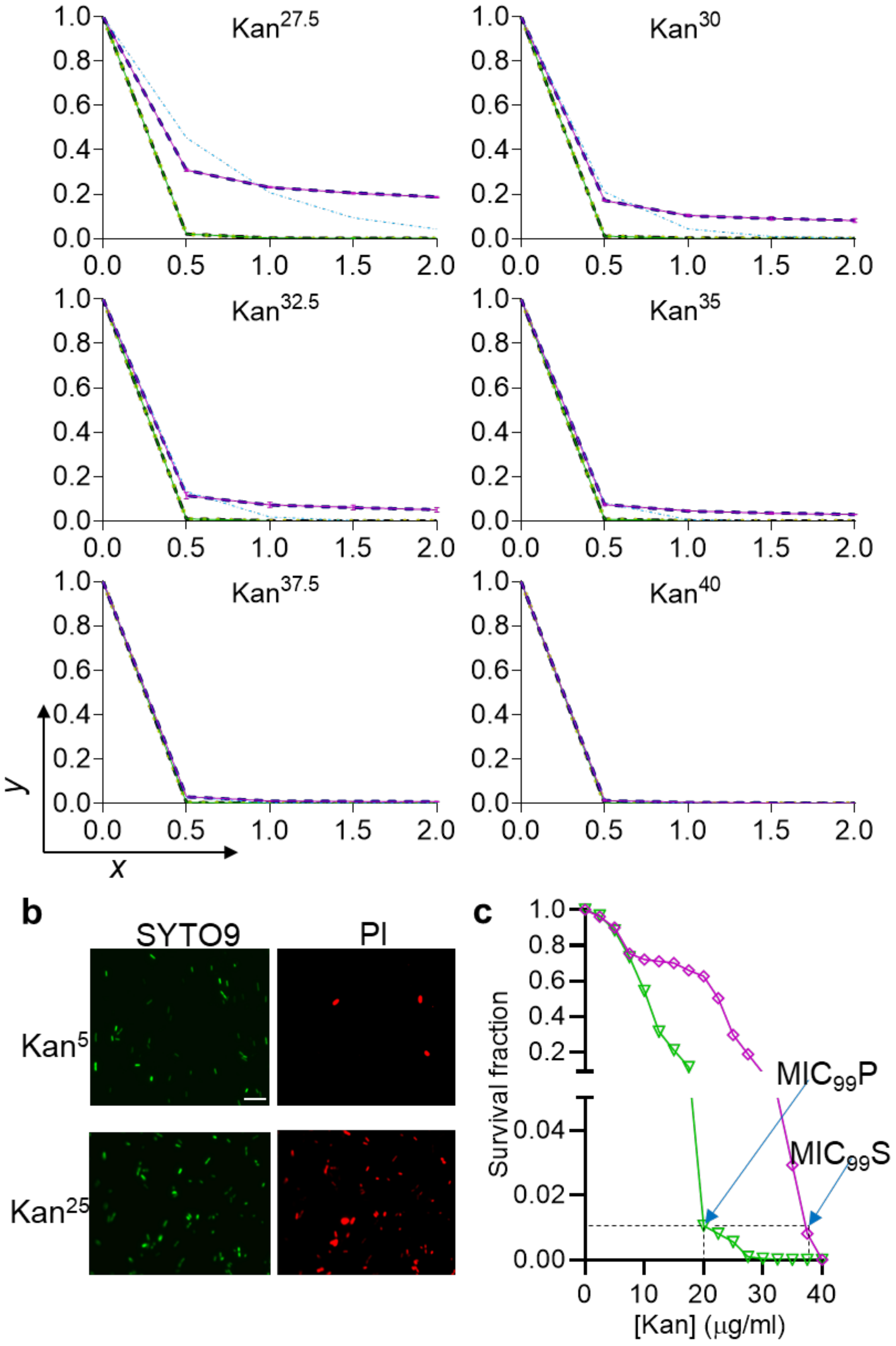

Supplementary Figure 1. Experimental and simulated kanamycin survival curves a. Survival curves of planktonic versus swarm populations (solid lines) at kanamycin (Kan) concentrations ranging from 2.5 to

40 μg/ml. The superscript following Kan refers to μg/ml. Time in hours is plotted on the ***x*** axis, and fraction survived (as determined by CFU counts) on the ***y*** axis. Multiple unpaired t-tests, with statistical significance determined using the Holm-Sidak method (with alpha = 0.05), were performed on all experimental data points (see Supplementary Table 2 for p values); at early time points (0.5 h) in $Kan^{2.5}$-Kan^{35}, p values are significant and have a range of *p<0.05 to **p<0.001. The experimental data was simulated assuming either heterogeneous or a homogeneous planktonic or swarm populations, as described in Methods, and the best fit of the simulation to the experimental data is shown (dotted lines). See inset at the top right for legend keys. Graphs showing the distribution of the mean squared errors (MSE) between simulated and experimental data sets are shown in Figure 1a. **b.** Representative Live-Dead staining images of swarm cells treated with Kan^{5} and Kan^{25} for 0.5 h, confirming the CFU count data in **a**. All cells are stained with the green dye SYTO9, but only cells with membrane damage (dead cells) stain with the red dye propidium iodide (PI). Scale bar, 10 μm **c.** Determination of MIC (minimum inhibitory concentration) for Kan from survival curves at 2 h shown in **a**. MIC_{99}, the antibiotic concentration required to kill 99% of the population is Kan^{19} for planktonic cells (green, $MIC_{99}P$), and $Kan^{37.5}$ for swarm cells (red, $MIC_{99}S$). Source data are provided as a Source Data file.

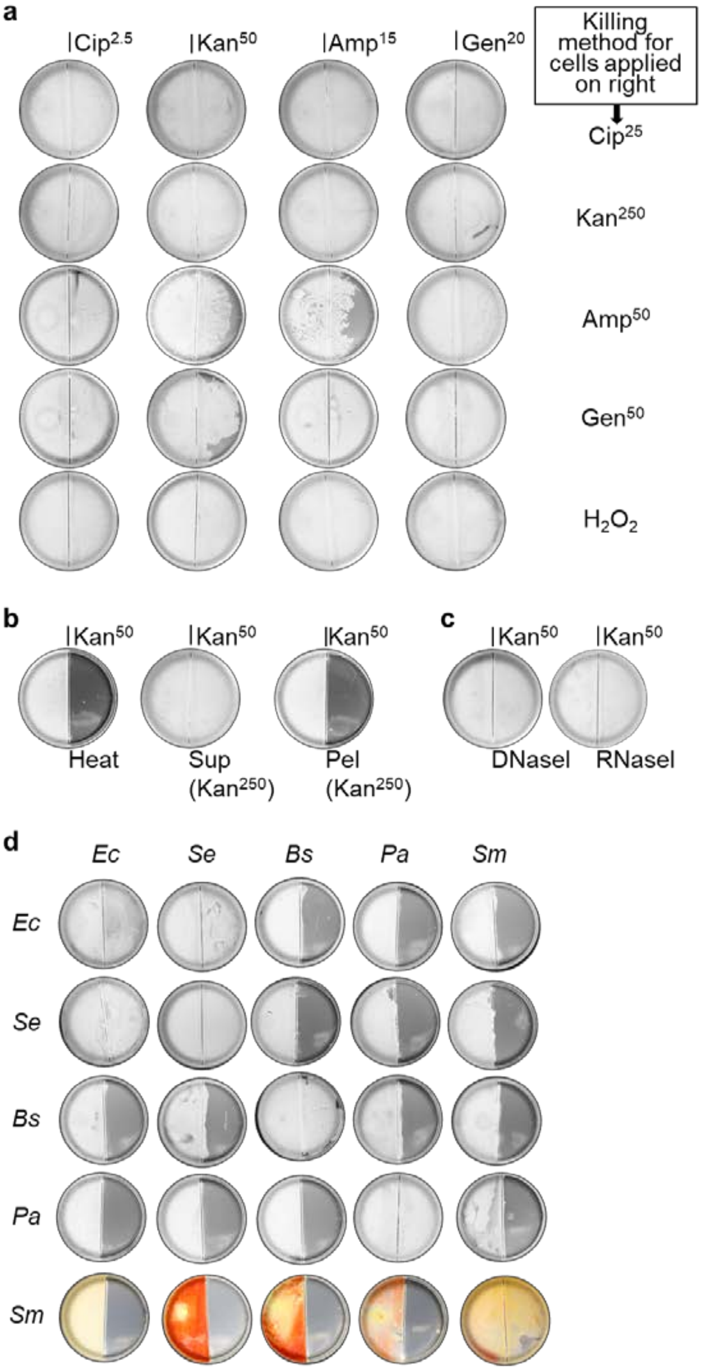

Supplementary Figure 2. Characteristics of the necrosignal. a. Dead cell-promoted SR is independent of the killing method. *E. coli* swarms are sensitive to Cip$^{2.5}$, Kan^{25}, Amp^{15} and Gen^{20}. Wild type *E. coli* cells were inoculated on the left, and cells killed by indicated methods were applied on the right. Antibiotics applied to the right are indicated. **b.** The necrosignal is heat-sensitive and found in the supernatant fraction of cell extracts from killed *E. coli*. Heat killed cells (Heat) or supernatant (Sup) and pellet (Pel) fractions from cells killed with Kan^{250}, were applied to the right to assess swarming on Kan^{50}. **c**. The necrosignal is not DNA or RNA. Supernatant from cells killed with Kan^{250} were treated with DNaseI and RNaseI and applied on the right. **d.** Species-specificity of necrosignaling. This panel shows the raw data for results summarized in Figure 1e. The right chamber contained Kan^{50} for *Ec*, *Se*, and *Bs*; Kan^{75} for *Sm*; Kan^{125} for *Pa*. Except for *Pseudomonas*, which was pre-killed by Kan^{500}, all others were pre-killed Kan^{250}*S. marcescens* makes a red pigment in stationary phase[1].

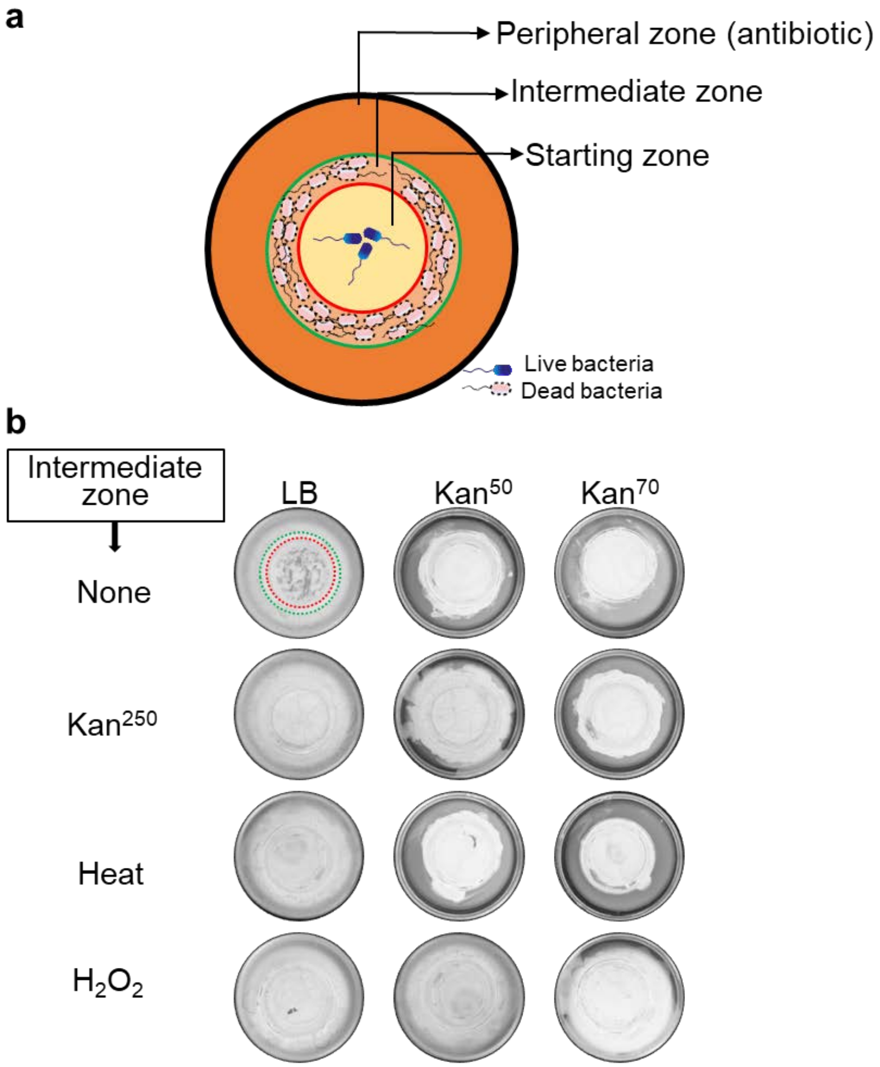

Supplementary Figure 3. Tri-plate assay showing the sustained nature of the SR response. a. Cartoon demonstrating placement of plates of different diameters inside one another to create three swarming zones as labeled, with narrow agar bridges connecting the chambers as described in Figure 2a. **b.** WT *E. coli* was inoculated in the starting zone in all plates. The composition of cells deposited on the intermediate zone is indicated on the left. The presence and concentration of antibiotics in the peripheral zone is indicated above each row of plates. The data show that cells swarming over the intermediate dead cell zone are potentiated for SR even after exiting this zone, as seen by their ability to swarm on the peripheral antibiotic zone. This response is most pronounced with 50 mM H_2O_2, promoting swarming on Kan^{70} in the peripheral zone. Heat destroys the response. Dotted red and green circles on the top left tri-plate show outlines of smallest and medium sized plates, respectively.

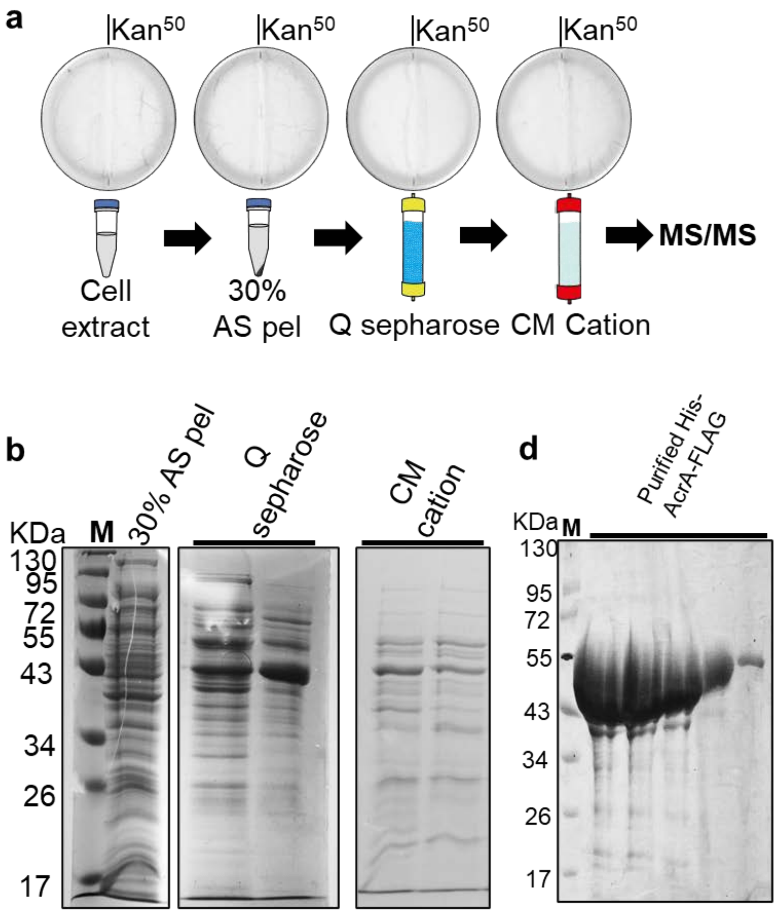

c

Step	Minimum protein required for activity (mg)	Fold purification
Cell extract	12.7	1
30% pellet	2.194	5.78
Q sepharose	0.119	106.72
CM cation	0.073	173.97

Supplementary Figure 4. Purification of the necrosignal from *E. coli* and *Salmonella*. a. Necrosignaling activity in cell extract supernatants was precipitated with ammonium sulfate (AS pel), resuspended and fractionated over Q Sepharose and CM Cation exchange columns, and analyzed by MS/MS. See Methods for purification details. Plates shown are for *E. coli.* **b.** 12% SDS-PAGE gels showing *E. coli* active fractions from 30% AS pel, Q Sepharose (fractions 5 and 6) and CM cation (fractions 1 and 2); M, protein MW markers. **c**. Table showing fold-purification achieved in *E. coli*. **d.** Gel from 12% SDS-PAGE showing purification of His- and FLAG-tagged AcrA. From left to right, fractions 1-5 from a 5-step imidazole gradient elution (see Methods). 10 μl of each fraction was loaded on the gel. Source data are provided as a Source Data file.

Escherichia coli			
Uniprot ID	Protein description	Name	MW (kDa)
P06959	Pyruvate dehydrogenase, E2 subunit	AceF	66
P0AE06	Multidrug efflux pump subunit AcrA	AcrA	42
P69228	DNA-binding transcriptional regulator BaeR	BaeR	21
P0ACJ8	DNA-binding transcriptional dual regulator CRP	Crp	24
P0A6P9	Enolase	Eno	46
P0A9X4	Dynamic cytoskeletal protein MreB	MreB	37
P23836	DNA-binding transcriptional dual regulator PhoP	PhoP	26
P0ADY1	Periplasmic folding chaperone	PpiD	68
P0CG19	truncated RNase PH	Rph	24
P0A7V8	30S ribosomal protein S4	RpsD	23
P0AAC0	universal stress protein	UspE	21
P0AFP4	NADP+-dependent aldehyde reductase	YbbO	29
P77316	Zinc-type alcohol dehydrogenase-like protein YbdR	YbdR	44
P0A8W8	UPF0304 protein YfbU	YfbU	20
P76550	uncharacterized protein YffS	YffS	30
P0ADT8	Uncharacterized protein	YgiM	23
P0A742	putative transporter	YhdC	43
P64429	uncharacterized protein YpfJ	YpfJ	31

Salmonella Enterica 14028			
Uniprot ID	Protein description	Name	MW (kDa)
Q8ZRA6	Multidrug efflux pump subunit AcrA	AcrA	42
Q8ZK33	Arginine deiminase	ArcA	46
Q8ZNP8	DNA-binding transcriptional regulator	BaeR	27
P0A2T6	DNA-binding transcriptional dual regulator CRP	Crp	27
Q8ZQD3	Anaerobic dimethyl sulfoxide reductase, subunit A	DmsA	90
P06179	Flagellin protein	FliC	51
Q8ZRU0	Cell division protein	FtsZ	40
P0A1H3	Traslation elongation factor G	FusA	77
Q8ZNG5	Glycerol-3-phosphate dehydrogenase subunit A	GlpA	59
Q8ZLH4	Glycerol-3-phosphate dehydrogenase	GlpD	56
Q8ZN59	Inosine-5'-monophosphate dehydrogenase	GuaB	51
Q8ZN40	Cysteine desulfurase IscS	IscS	45
Q8ZRT0	Dihydrolipoyl dehydrogenase	LpdA	50
Q9RCI2	tRNA-2-methylthio-N(6)-dimethylallyladenosine synthase	MiaB	53
Q8ZP10	Septum site-determining protein	MinD	29
Q8ZLJ7	Transpeptidase of penicillin-binding protein 1a	MrcA	94
P41033	Phosphoenolpyruvate carboxykinase	PckA	59
Q8ZK80	50S ribosomal protein L9	RplI	15
Q8ZRT7	Protein translocase subunit SecA	SecA	101
Q8ZP84	Universal stress protein	UspE	35
Q8ZPP0	Inner membrane transport protein	YdhC	42

Supplementary Figure 5. MS/MS analysis of the SR-promoting active fraction from *E. coli* and *Salmonella*. The active fractions analyzed were obtained from the CM cation step of purification. See Supplementary Figure 4 and Methods. 19 proteins were identified for *E. coli* and 21 proteins for *Salmonella*; of these, 5 proteins were common.

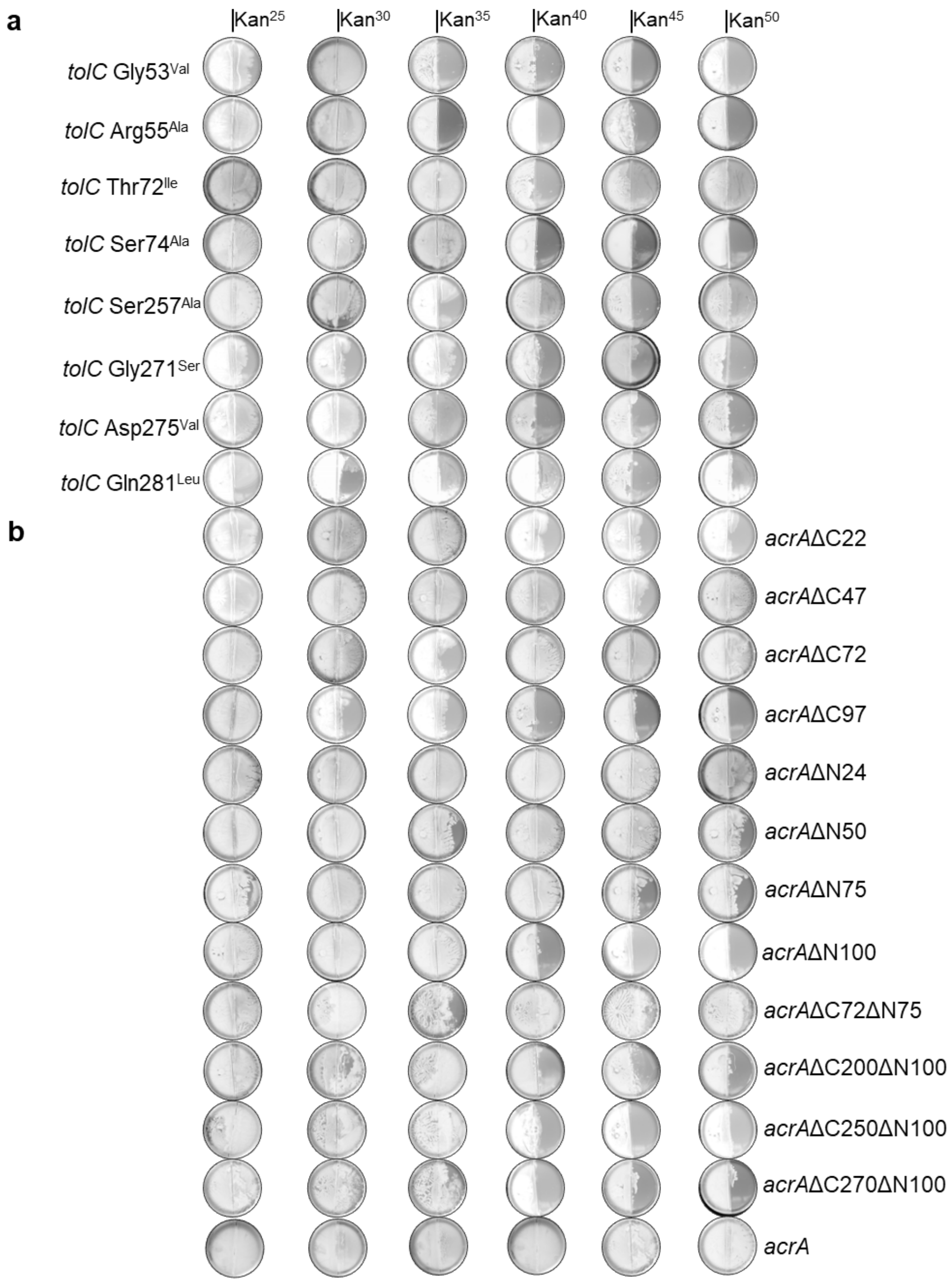

Supplementary Figure 6. Primary data for results summarized in Fig 4c. a. Border crossing assay with *tolC* mutants. The Δ*tolC*$^{\Delta K}$ strain with indicated mutants were inoculated in the left chamber and purified AcrA was applied on the right. The WT control for this panel is found in Figure 4, a2. **b.** Border crossing assay with *acrA* mutants. WT cells were inoculated on the left and pre-killed cells (Δ*acrA*$^{\Delta K}$) expressing indicated *acrA* mutants from pTrc99a plasmids were applied on the right. Indicated Kan concentrations are for the right chamber.

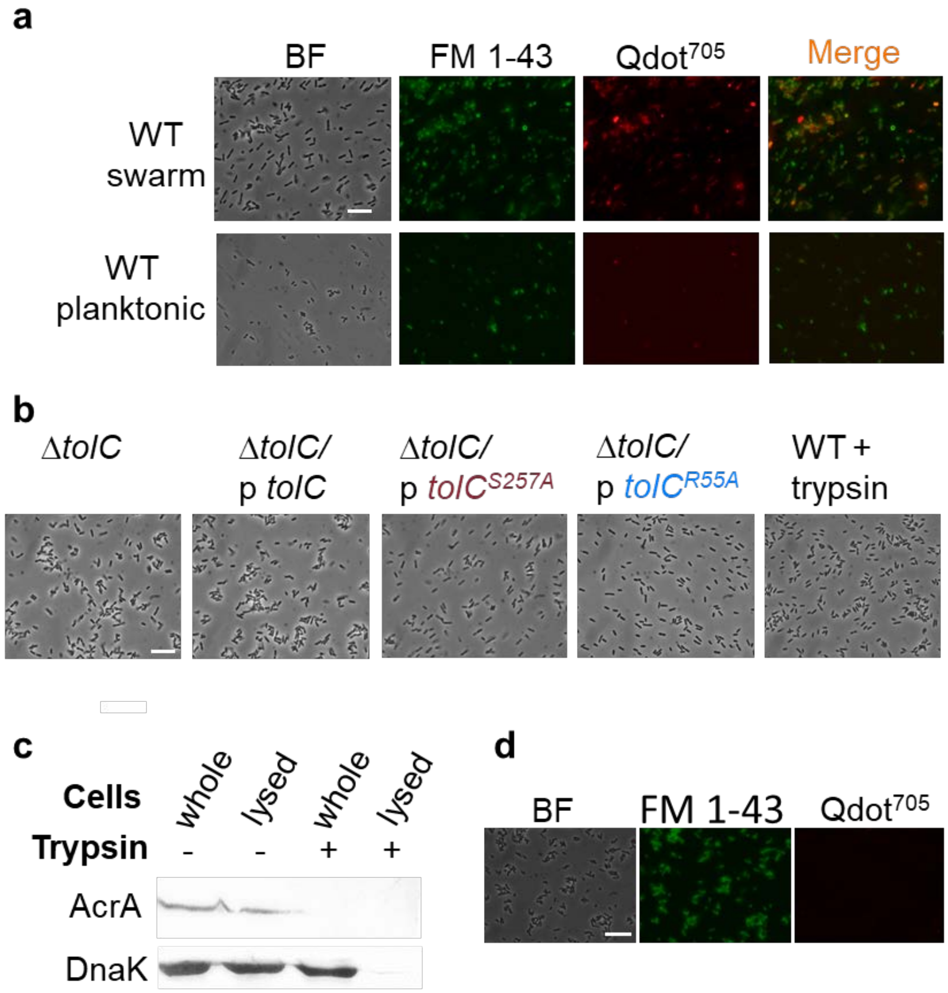

Supplementary Figure 7. **Binding of extracellular AcrA to TolC on planktonic and swarm cells of *E. coli***. **a.** Imaging of cells probed with QDot^{705} anti-FLAG antibody and FM-143 (see Methods). The entire field of view (for each channel under the microscope) of the images that were enlarged in Figure 4d, are observable here. Swarm cells are approximately twice as long as planktonic cells[2]. Scale bar, 10 μm. **b**. Bright field images of swarm cells shown in Figure 4e. *tolC* mutant font color Blue = SR+, maroon = SR-. **c.** Western blot analysis for detecting AcrA localization after trypsin digestion of cell samples used for microscopy in Figure 4d (see Methods). DnaK was used as a cytoplasmic control. Absence of bands for AcrA (FLAG-tagged) from whole cells treated with trypsin indicate extracellular localization of AcrA in the binding experiment shown in (**a**). **d.** Additional control showing that Qdot^{705} alone, i.e. without addition of AcrA-FLAG protein, does not localize to membrane. Source data are provided as a Source Data file.

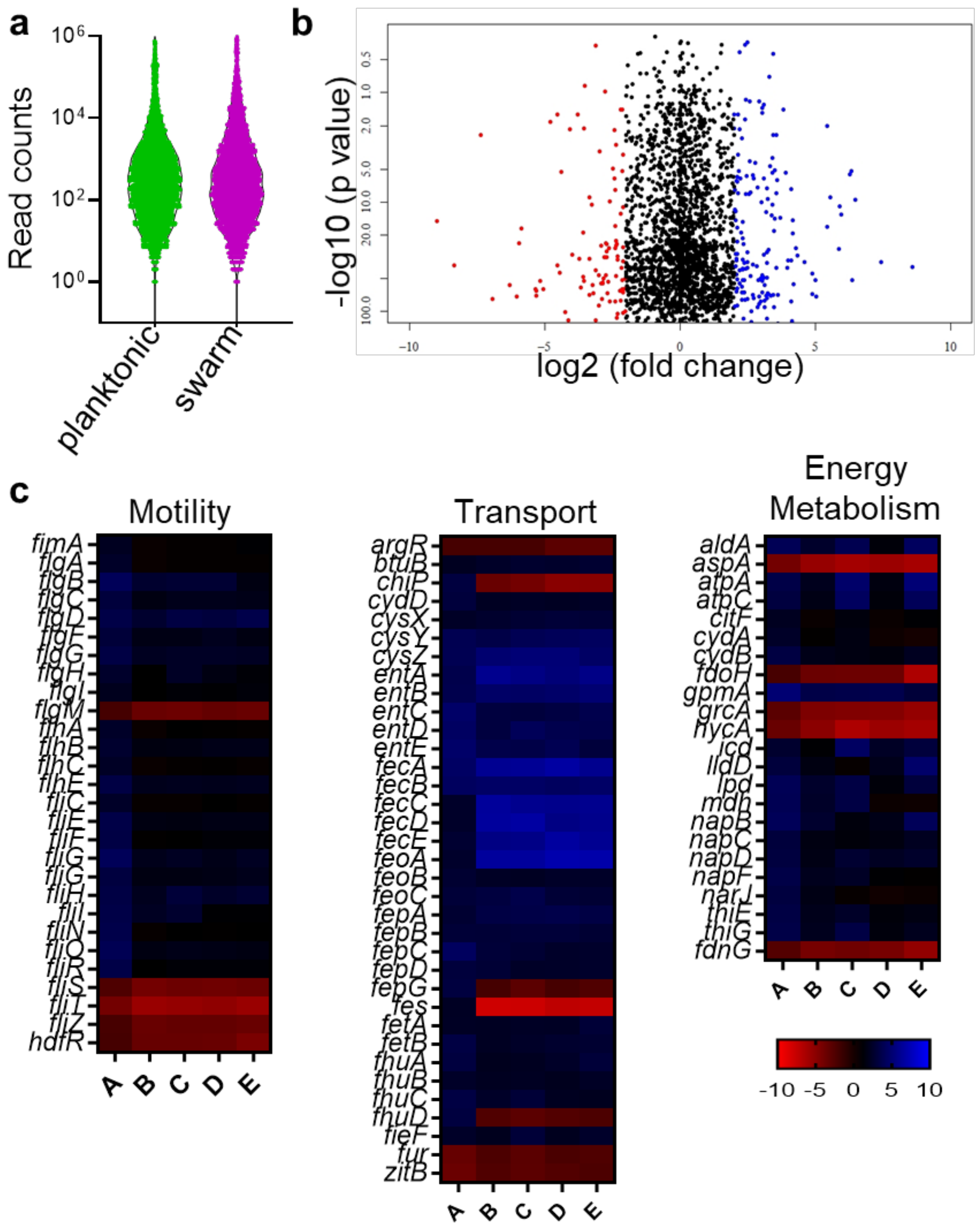

Supplementary Figure 8. RNA-seq analysis of swarm and planktonic cells of *E. coli*. a. Distribution of mapped read counts for genes showing sequencing consistency across samples. **b.** A representative scatter plot showing fold change and adjusted p-value distributions of genes for swarm vs planktonic. A ±2 log2 fold change cutoff was used to identify up or downregulated genes. The blue and red dots represent log2 fold change with a value of >= 2 and <=-2, respectively. **c.** Heat maps showing changes in expression of the different gene classes (motility, transport, energy metabolism) across the following comparisons: A) Swarm vs Planktonic, B) Swarm vs Swarm+ Kan^{20}, C) Swarm vs Swarm+Kan^{20}+AcrA (0.1 μg/ml), D) Swarm vs Swarm+$Cip^{2.5}$, E) Swarm vs Swarm+ $Cip^{2.5}$+AcrA (0.1 μg/ml). See Supplementary Data 1 for expression values. Source data are provided as a Source Data file.

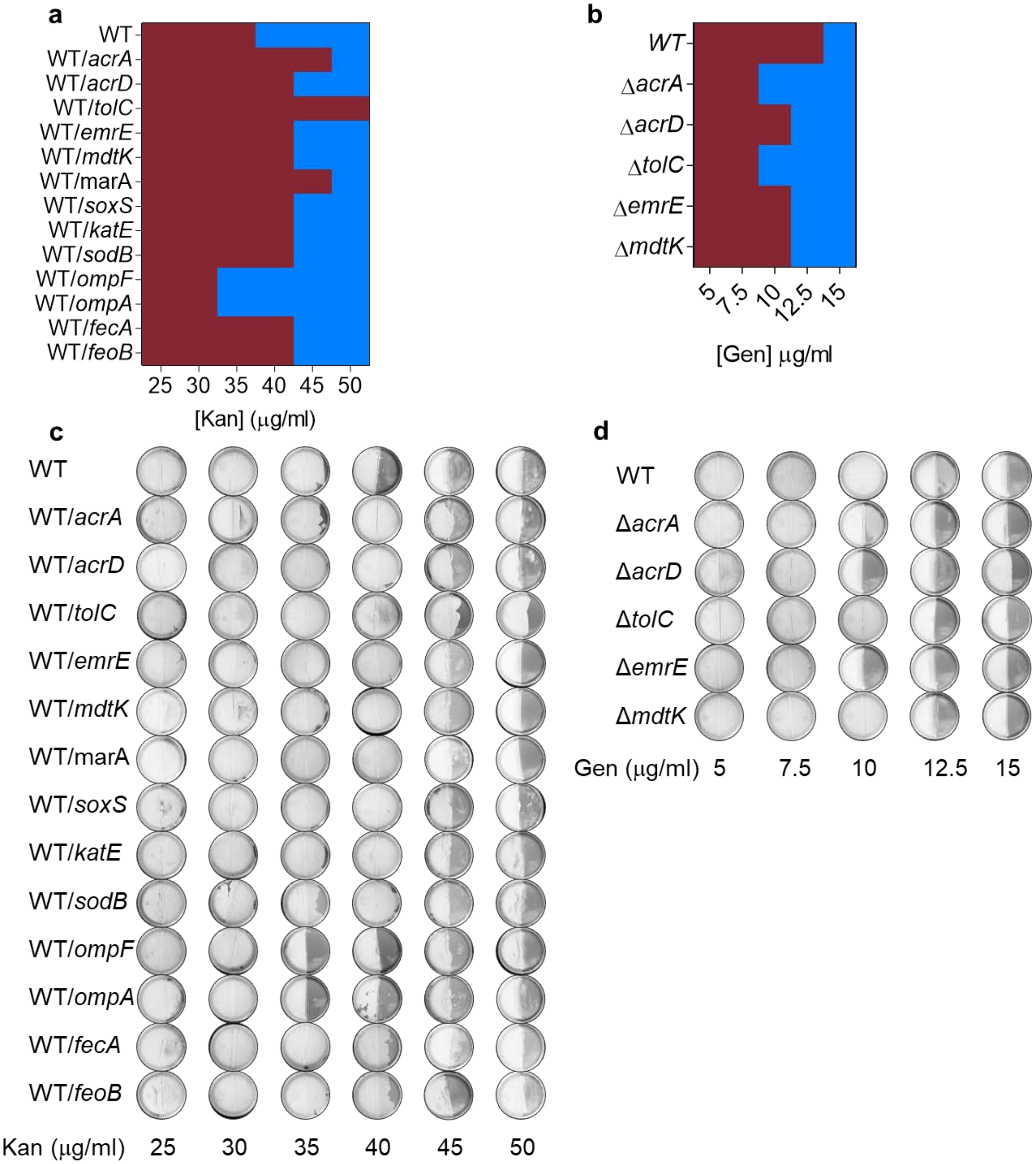

Supplementary Figure 9. Validation of RNA-seq results through genetic analysis. Summary of border crossing assays. Blue, SR+; Maroon, SR-. WT *E. coli* carrying plasmids from ASKA library or gene deletions were inoculated on the left. **a**. The overexpression of selected components from different classes of efflux pumps (*acrA, acrD, tolC, emrE, mdtk*) and their regulators (*marA* and *soxS*), ROS catabolism genes (*katE* and *sodB*), and iron transport genes (*fepA and feoB*) showed an increase in SR. Porins *ompF* and *ompA* reduced SR. **b.** Deletion strains of selected efflux pump genes (*acrA*, *acrD*, *tolC*, *mdtK*, and *emrE*), inoculated on the left, reduced SR. Gentamycin [Gen] was used in the right chamber as these strains harbor a Kan^{R} marker. **c-d.** Raw data for **a** and **b** respectively.

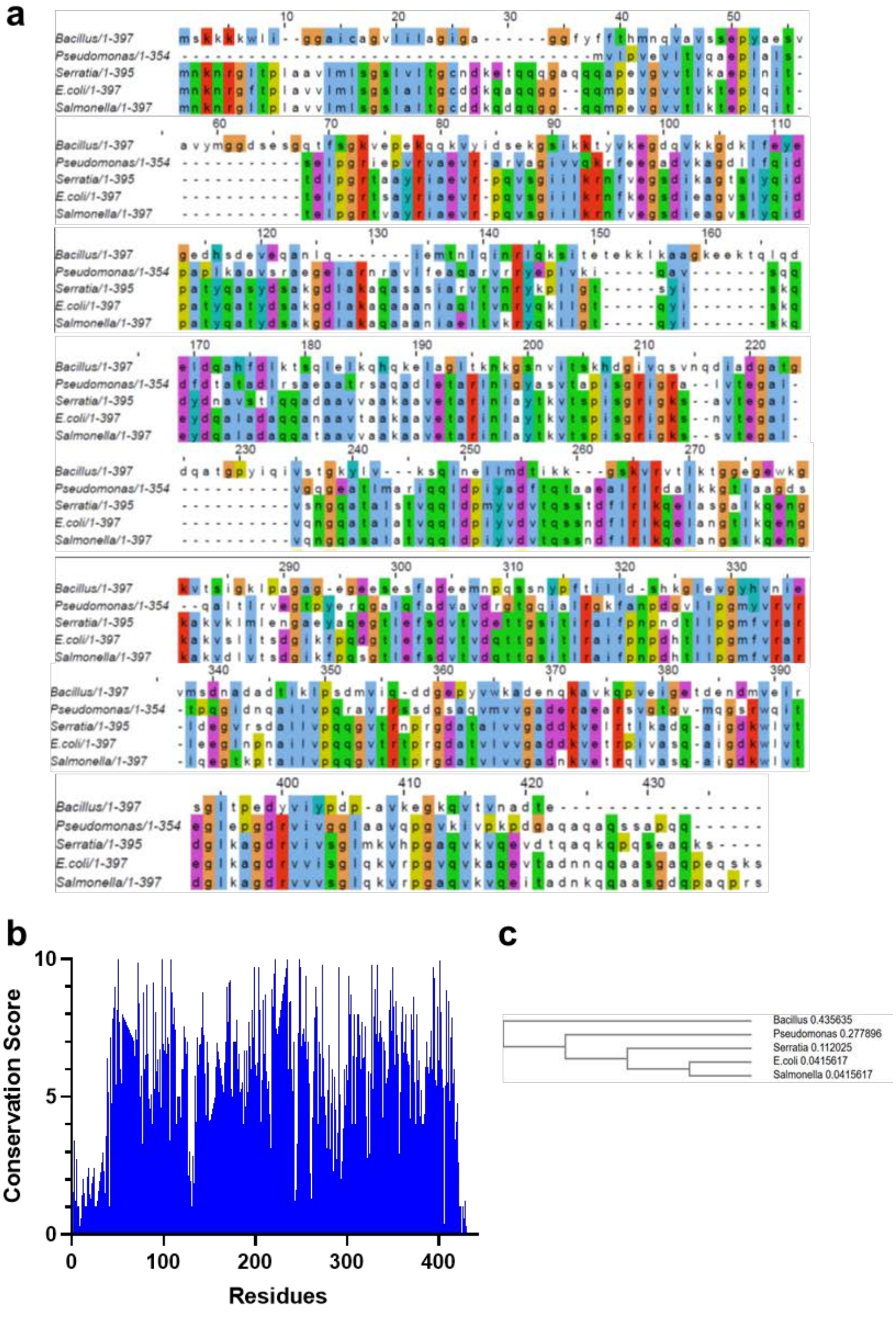

Supplementary Figure 10. Comparison of AcrA sequences in five organisms used in this study. a. Multiple Sequence Alignment (MSA) of AcrA sequences (see Methods). **b.** Graph showing variations in conservation scores across residues; numbering relates to *E. coli* AcrA sequence. **c.** A cladogram of AcrA sequences.

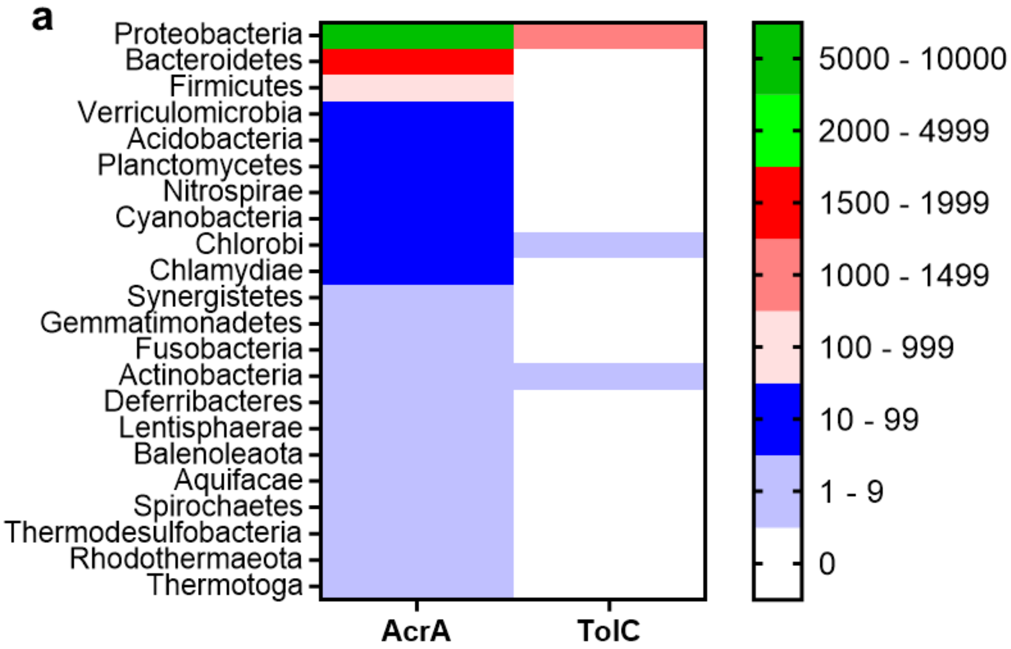

Supplementary Figure 11. Distribution of AcrA and TolC across the Bacterial domain. The sequences of AcrA and TolC of *E. coli* were used to perform a search using HMMER algorithm[3] within bacterial taxa. The significant 'e value' cutoff was 10^{-20}. A decrease or increase in the cutoff (10^{-10} to 10^{-3}) did not alter the pattern of results significantly. The values show the number of hits obtained across different taxa.

Supplementary Tables

Supplementary Table 1. Strains, Plasmids, and Primers

Strain/Plasmid	Genotype	Source and/or reference
**Strains**		
Escherichia coli		
MG1655	Wild type; F^- λ^- *rph*-1	[4]
Δ*acrA*	MG1655 Δ*acrA::*Kan^R	This study
ΔacrA$^{\Delta K}$	MG1655 Δ*acrA, Kan*^{R} removed	
Δ*uspE*	MG1655 Δ*uspE::*Kan^R	
Δ*baeR*	MG1655 Δ*baeR::*Kan^R	
Δ*yhdC*	MG1655 Δ*yhdC::*Kan^R	
Δ*tolC*	MG1655 Δ*tolC::*Kan^R	
ΔtolC$^{\Delta K}$	MG1655 Δ*tolC, Kan*^{R} removed	
Δ*acrD*	MG1655 Δ*acrD::*Kan^R	
Δ*emrE*	MG1655 Δ*emrE::*Kan^R	
Δ*mdtK*	MG1655 Δ*mdtK::*Kan^R	
Salmonella enterica		
serovar Typhimurium 14028	Wild type; LT2 (*fljB-off*) Δ(*hin-fljAB*)	ATCC
Serratia marcescens		
274	Wild type	ATCC
Bacillus subtilis		
3610	Motile wild type	[5]
Pseudomonas aeruginosa		
PAO1	Wild type	Lab collection
**Plasmids**		
pCA24N	From ASKA collection, Cam^R	ASKA[6]
p*acrA*	pCA24N carrying *acrA*	
p*uspE*	pCA24N carrying *uspE*	
p*baeR*	pCA24N carrying *baeR*	
p*yhdC*	pCA24N carrying *yhdC*	
p*acrD*	pCA24N carrying *acrD*	
p*tolC*	pCA24N carrying *tolC*	
p*emrE*	pCA24N carrying *emrE*	
p*mdtK*	pCA24N carrying *mdtK*	
p*marA*	pCA24N carrying *marA*	
p*soxS*	pCA24N carrying *soxS*	
p*katE*	pCA24N carrying *katE*	
p*sodB*	pCA24N carrying *sodB*	
p*ompF*	pCA24N carrying *ompF*	
p*ompA*	pCA24N carrying *ompA*	

p*fecA*	pCA24N carrying *fecA*	
p*feoB*	pCA24N carrying *feoB*	
pTrc99a	Cloning vector, P_{Trc} and Amp^R,	[7]
pT*tolC*	pTrc99a carrying WT *tolC*	This study
pT*tolC* $Gly53^{Val}$	pTrc99a carrying mutant *tolC* $Gly53^{Val}$	
pT*tolC* $Arg55^{Leu}$	pTrc99a carrying mutant *tolC* $Arg55^{Leu}$	
pT*tolC* $Thr72^{Ile}$	pTrc99a carrying mutant *tolC* $Thr72^{Ile}$	
pT*tolC* $Ser74^{Ala}$	pTrc99a carrying mutant *tolC* $Ser74^{Ala}$	
pT*tolC* $Ser257^{Ala}$	pTrc99a carrying mutant *tolC* $Ser257^{Ala}$	
pT*tolC* $Gly271^{Ser}$	pTrc99a carrying mutant *tolC* $Gly271^{Ser}$	
pT*tolC* $Asp275^{Val}$	pTrc99a carrying mutant *tolC* $Asp275^{Val}$	
pT*tolC* $Gln281^{Leu}$	pTrc99a carrying mutant *tolC* $Gln281^{Leu}$	
pT*acrA*	pTrc99a carrying WT *acrA*	
pT*acrA*ΔC22	pTrc99a carrying mutant *acrA*ΔC22	
pT*acrA*ΔC47	pTrc99a carrying mutant *acrA*ΔC47	
pT*acrA*ΔC72	pTrc99a carrying mutant *acrA*ΔC72	
pT*acrA*ΔC97	pTrc99a carrying mutant *acrA*ΔC97	
pT*acrA*ΔN24	pTrc99a carrying mutant *acrA*ΔN24	
pT*acrA*ΔN50	pTrc99a carrying mutant *acrA*ΔN50	
pT*acrA*ΔN75	pTrc99a carrying mutant *acrA*ΔN75	
pT*acrA*ΔN100	pTrc99a carrying mutant *acrA*ΔN100	
pT*acrA*ΔC72ΔN75	pTrc99a carrying mutant *acrA*ΔC72ΔN75	
pT*acrA*ΔC200ΔN100	pTrc99a carrying mutant *acrA*ΔC200ΔN100	
pT*acrA*ΔC250ΔN100	pTrc99a carrying mutant *acrA*ΔC250ΔN100	
pTacrA ΔC270ΔN100	pTrc99a carrying mutant *acrA*ΔC270ΔN100	

**Primers**	
**Name**	**Sequence (5’→3’)**
GLY53 Fp	ACCTATAGCAACGTCTACCGCGACGCGAACGGCATCAACT
GLY53 Rp	CGCGTCGCGGTAGACGTTGCTATAGGTGTAATCTGCACCT
ARG55 Fp	AGCAACGGCTACGCGGACGCGAACGGCATCAACTCT
ARG55 Rp	GCCGTTCGCGTCCGCGTAGCCGTTGCTATAGGTGTA
THR72 Fp	TCCTTGCAGTTAATTCAATCCATTTTTGATATGTCGAAATGG
THR72 Rp	AAAAATGGATTGAATTAACTGCAAGGACGCACTGGTCGCG
SER74 Fp	CAGTTAACTCAAGCCATTTTTGATATGTCGAAATGGCGTGCG
SER74 Rp	CATATCAAAAATGGCTTGAGTTAACTGCAAGGACGCACTGGT
SER257 Fp	TCTACCGGGATTGCTGACACCTCTTATAGCGGTTCGAAAACC
SER257 Rp	ATAAGAGGTGTCAGCAATCCCGGTAGAAGCCGTTAAATCCAG
GLY271 Fp	CGTGGTGCCGCTAGTACCCAGTATGACGATAGCAATATGGGC
GLY271 Rp	GTCATACTGGGTACTAGCGGCACCACGGGTTTTCGAACCGCT
ASP275 Fp	GGTACCCAGTATGTCGATAGCAATATGGGCCAGAACAAAGTT
ASP 275 Rp	CATATTGCTATCGACATACTGGGTACCAGCGGCACCACGGGT
GLN281 Fp	AGCAATATGGGCCTGAACAAAGTTGGCCTGAGCTTCTCGCTG
GLN281 Rp	GCCAACTTTGTTCAGGCCCATATTGCTATCGTCATACTGGGT
acrA Fp	CGAGGTGGATCCATGAACAAAAAC
acrA Rp	GCTCAAGCTTAAGTTAAGACTTG

acrADC22 Rp	TGCTTTTACCTGGACACCAGGTAAAAGCTTAGAT
acrADC47 Rp	TGTCACCAGCCACTTATCGCCTAAAAGCTTAGAT
acrADC72 Rp	TACCAGTACGGTGGCATCGCCTAAAAGCTTAGAT
acrADC97 Rp	CCCTTCTTCCAGACGTGCGCGTAAAAGCTTAGAT
acrADC200 Rp	GTTCTGTACCAATGCGCCTTCTAAAAGCTTAGAT
acrADC250 Rp	CAGAGCCTGATCGTACTCTTGTAAAAGCTTAGAT
acrADC270 Rp	ATTCACCGTCAATTGCGCGATTAAAAGCTTAGAT
acrADN24 Fp	GTACGGATCCATGTGTGACGACAAACAGGCCCAA
acrADN50 Fp	GTACGGATCCATGCAGATCACAACCGAGCTTCCG
acrADN75 Fp	GTACGGATCCATGATCCTGAAGCGTAATTTCAAA
acrADN100 Fp	GTACGGATCCATGTATCAGGCGACATACGACAGT
arcA N ter His Fp	CATGAGCTCATGCATCATCATCATCATCACTGTGACGACAAA
acrA C-ter FLAG Rp	TTAAGCTTAGTCGTCATCGTCTTTGTAGTCAGACTTGGACTGTTC

Supplementary Table 2. Results of Statistical analysis of data in Supplementary Figure 1a

See **Supplementary Figure** 1 legend for details.

	p value (swarm experimental vs planktonic experimental)			
**Kan Concentration**	**0.5 h**	**1 h**	**1.5 h**	**2 h**
2.5	0.015258	0.050506	0.696204	0.728697
5	0.0173	0.01771	0.347403	0.07399
7.5	0.01991	0.049421	0.34826	0.079605
10	0.04439	0.149273	0.03715	0.000069
12.5	0.025151	0.044125	0.000353	0.000009
15	0.015377	0.000055	0.000016	0.000011
17.5	<0.000001	<0.000001	<0.000001	<0.000001
20	0.000007	<0.000001	<0.000001	<0.000001
22.5	0.000004	<0.000001	<0.000001	<0.000001
25	<0.000001	<0.000001	<0.000001	<0.000001
27.5	<0.000001	<0.000001	<0.000001	<0.000001
30	0.00216	0.000005	0.000047	0.000095
32.5	0.00229	0.00514	0.0059	0.00893
35	0.02494	0.138682	0.24732	0.34833
37.5	0.078213	0.18495	0.293395	0.112014
40	0.164641	<0.13922	0.127767	0.072741
[ns= not significant, *$p < 0.05$, **$p < 0.01$, and ***$p < 0.001$]				

References

1 Williams, R. P., Gott, C. L., Qadri, S. M. & Scott, R. H. Influence of temperature of incubation and type of growth medium on pigmentation in Serratia marcescens. *Journal of bacteriology* **106**, 438-443 (1971).

2 Partridge, J. D. & Harshey, R. M. More than motility: Salmonella flagella contribute to overriding friction and facilitating colony hydration during swarming. *Journal of bacteriology* **195**, 919-929, doi:10.1128/JB.02064-12 (2013).

3 Potter, S. C. *et al.* HMMER web server: 2018 update. *Nucleic acids research* **46**, W200-W204, doi:10.1093/nar/gky448 (2018).

4 Guyer, M. S., Reed, R. R., Steitz, J. A. & Low, K. B. Identification of a sex-factor-affinity site in E. coli as gamma delta. *Cold Spring Harbor symposia on quantitative biology* **45 Pt 1**, 135-140, doi:10.1101/sqb.1981.045.01.022 (1981).

5 Kearns, D. B. & Losick, R. Swarming motility in undomesticated Bacillus subtilis. *Molecular microbiology* **49**, 581-590, doi:10.1046/j.1365-2958.2003.03584.x (2003).

6 Kitagawa, M. *et al.* Complete set of ORF clones of Escherichia coli ASKA library (a complete set of E. coli K-12 ORF archive): unique resources for biological research. *DNA research : an international journal for rapid publication of reports on genes and genomes* **12**, 291-299, doi:10.1093/dnares/dsi012 (2005).

7 Amann, E., Brosius, J. & Ptashne, M. Vectors bearing a hybrid trp-lac promoter useful for regulated expression of cloned genes in Escherichia coli. *Gene* **25**, 167-178, doi:10.1016/0378-1119(83)90222-6 (1983).
