# Supplementary material for: Dead cells release a ‘necrosignal’ that activates antibiotic survival pathways in bacterial swarms
Source: Nat Commun. 2020 Aug 19;11:4157. doi: 10.1038/s41467-020-17709-0 (PMC7438516; doi:10.1038/s41467-020-17709-0)
Supplement: Supplementary file 3 — Reporting Summary [file 41467_2020_17709_MOESM3_ESM.pdf]

nature research

Corresponding author(s): Rasika Harshey

Last updated by author(s): Jun 23, 2020

Reporting Summary

Nature Research wishes to improve the reproducibility of the work that we publish. This form provides structure for consistency and transparency in reporting. For further information on Nature Research policies, see our Editorial Policies and the Editorial Policy Checklist.

Statistics

For all statistical analyses, confirm that the following items are present in the figure legend, table legend, main text, or Methods section.

n/a	Confirmed	
[ ]	[x]	The exact sample size (*n*) for each experimental group/condition, given as a discrete number and unit of measurement
[ ]	[x]	A statement on whether measurements were taken from distinct samples or whether the same sample was measured repeatedly
[ ]	[x]	The statistical test(s) used AND whether they are one- or two-sided<br>*Only common tests should be described solely by name; describe more complex techniques in the Methods section.*
[x]	[ ]	A description of all covariates tested
[ ]	[x]	A description of any assumptions or corrections, such as tests of normality and adjustment for multiple comparisons
[ ]	[x]	A full description of the statistical parameters including central tendency (e.g. means) or other basic estimates (e.g. regression coefficient) AND variation (e.g. standard deviation) or associated estimates of uncertainty (e.g. confidence intervals)
[ ]	[x]	For null hypothesis testing, the test statistic (e.g. *F*, *t*, *r*) with confidence intervals, effect sizes, degrees of freedom and *P* value noted<br>*Give P values as exact values whenever suitable.*
[x]	[ ]	For Bayesian analysis, information on the choice of priors and Markov chain Monte Carlo settings
[x]	[ ]	For hierarchical and complex designs, identification of the appropriate level for tests and full reporting of outcomes
[x]	[ ]	Estimates of effect sizes (e.g. Cohen's *d*, Pearson's *r*), indicating how they were calculated

Our web collection on statistics for biologists contains articles on many of the points above.

Software and code

Policy information about availability of computer code

Data collection	Proteomics: Sequest1.4, X! Tandem (Cyclone 2010)<br>Microscopy: Olympus CellSens 1.6<br>RNA Seq: Illumina Platform Nextseq 500
Data analysis	Data presentation and Statistical Analysis: Graphpad8, Microsoft Excel 2016, R Version 4.0.1, R studio 1.3<br>Image Processing and presentation: Adobe Photoshop CC2017, Adobe Illustrator CS6, Microsoft Powerpoint 2016<br>Proteomics: Scaffold 4.6<br>RNA Seq: FastQC v0.13, MultiQC 1.7, Cutadapt 1.9, Bowtie2 v2.3.5, Samtools 1.8, Bedtools 2.27, DESeq2 3.8, DAVID 6.8<br>Protein-Protein Interaction: HADDOCK 2.2<br>Protein sequence analysis: Jalview 2.10, HMMER 3.2, Clustal Omega 1.2<br>Protein Structure Analysis: Chimera 1.13

For manuscripts utilizing custom algorithms or software that are central to the research but not yet described in published literature, software must be made available to editors and reviewers. We strongly encourage code deposition in a community repository (e.g. GitHub). See the Nature Research guidelines for submitting code & software for further information.

Data

Policy information about availability of data

All manuscripts must include a data availability statement. This statement should provide the following information, where applicable:

- Accession codes, unique identifiers, or web links for publicly available datasets
- A list of figures that have associated raw data
- A description of any restrictions on data availability

The RNA seq data was deposited to SRA with BioProject ID PRJNA640755 [http://www.ncbi.nlm.nih.gov/bioproject/640755]. Source data are provided with this paper.

Field-specific reporting

Please select the one below that is the best fit for your research. If you are not sure, read the appropriate sections before making your selection.

- [x] Life sciences
- [ ] Behavioural & social sciences
- [ ] Ecological, evolutionary & environmental sciences

For a reference copy of the document with all sections, see nature.com/documents/nr-reporting-summary-flat.pdf

Life sciences study design

All studies must disclose on these points even when the disclosure is negative.

Sample size	Except for RNA seq (n=2), the sample size was equal to or larger than 3. No method was used to calculate the sample size as 3 biological replicates are the standards of this field.
Data exclusions	Data was not excluded from experiments, except for apparent failures.
Replication	All results of the experiments reported here were consistently reproduced across all biological replicates.
Randomization	Not applicable for this field of research as experiments were performed under well-controlled and defined conditions; also, no human/animal subjects were used in this study.
Blinding	Not applicable as there were no animal or human groups involved in our study, no blinding was performed.

Reporting for specific materials, systems and methods

We require information from authors about some types of materials, experimental systems and methods used in many studies. Here, indicate whether each material, system or method listed is relevant to your study. If you are not sure if a list item applies to your research, read the appropriate section before selecting a response.

Materials & experimental systems

n/a	Involved in the study	
[ ]	[x]	Antibodies
[x]	[ ]	Eukaryotic cell lines
[x]	[ ]	Palaeontology and archaeology
[x]	[ ]	Animals and other organisms
[x]	[ ]	Human research participants
[x]	[ ]	Clinical data
[x]	[ ]	Dual use research of concern

Methods

n/a	Involved in the study	
[x]	[ ]	ChIP-seq
[x]	[ ]	Flow cytometry
[x]	[ ]	MRI-based neuroimaging

Antibodies

Antibodies used	1. DYKDDDDK Tag monoclonal antibody (FGR4) (Thermo Fisher)<br>2. Qdot705-labeled anti-FLAG antibody<br>3. Anti-DnaK Antibody (abcam)

Validation

1.The following information was taken from manufacturer's website(https://www.thermofisher.com/antibody/product/DYKDDDDK-Tag-Antibody-clone-FG4R-Monoclonal/MA1-91878): This Antibody was verified by Cell treatment to ensure that the antibody binds to the antigen stated.
2. The antibody described above (1) was used to make the Qdot705-labeled anti-FLAG antibody by conjugating DYKDDDDK Tag antibody and Qdot705. It was validated by Western Blots and tested in Escherichia coli.
3. The following information was taken from manufacturer's website (https://www.abcam.com/dnak-antibody-8e22-ab69617.html): Validated by Western Blots and tested in Escherichia coli.
