# Supplementary material for: Dead cells release a ‘necrosignal’ that activates antibiotic survival pathways in bacterial swarms
Source: Nat Commun. 2020 Aug 19;11:4157. doi: 10.1038/s41467-020-17709-0 (PMC7438516; doi:10.1038/s41467-020-17709-0)
Supplement: Supplementary file 4 — Description of Additional Supplementary Files [file 41467_2020_17709_MOESM4_ESM.pdf]

Description of Additional Supplementary Files

Supplementary Data 1: Table showing comparison of expression values of genes from swarm and planktonic samples, represented as log2 fold change.
